# Automated detection and classification of the proximal humerus fracture by using deep learning algorithm

**DOI:** 10.1080/17453674.2018.1453714

**Published:** 2018-03-26

**Authors:** Seok Won Chung, Seung Seog Han, Ji Whan Lee, Kyung-Soo Oh, Na Ra Kim, Jong Pil Yoon, Joon Yub Kim, Sung Hoon Moon, Jieun Kwon, Hyo-Jin Lee, Young-Min Noh, Youngjun Kim

**Affiliations:** 1 Department of Orthopaedic Surgery and; 2 Department of Dermatology, I-dermatology clinic, Seoul;; 3 Department of Radiology, Konkuk University School of Medicine, Seoul;; 4 Department of Orthopaedic Surgery, Kyungpook National University College of Medicine, Daegu, Korea;; 5 Department of Orthopaedic Surgery, Myungji Hospital, Goyang;; 6 Department of Orthopaedic Surgery, Kangwon National University College of Medicine, Chuncheon, Korea;; 7 Department of Othopaedic Surgery, National Police Hospital, Seoul;; 8 Department of Orthopaedic Surgery, Catholic University College of Medicine, Seoul, St Mary’s Hospital, Seoul, Korea;; 9 Department of Orthopaedic Surgery, Dong-A University College of Medicine, Pusan;; 10 Center for Bionics, Korea Institute of Science and Technology, Seoul, Korea

## Abstract

Background and purpose — We aimed to evaluate the ability of artificial intelligence (a deep learning algorithm) to detect and classify proximal humerus fractures using plain anteroposterior shoulder radiographs.

Patients and methods — 1,891 images (1 image per person) of normal shoulders (n = 515) and 4 proximal humerus fracture types (greater tuberosity, 346; surgical neck, 514; 3-part, 269; 4-part, 247) classified by 3 specialists were evaluated. We trained a deep convolutional neural network (CNN) after augmentation of a training dataset. The ability of the CNN, as measured by top-1 accuracy, area under receiver operating characteristics curve (AUC), sensitivity/specificity, and Youden index, in comparison with humans (28 general physicians, 11 general orthopedists, and 19 orthopedists specialized in the shoulder) to detect and classify proximal humerus fractures was evaluated.

Results — The CNN showed a high performance of 96% top-1 accuracy, 1.00 AUC, 0.99/0.97 sensitivity/specificity, and 0.97 Youden index for distinguishing normal shoulders from proximal humerus fractures. In addition, the CNN showed promising results with 65–86% top-1 accuracy, 0.90–0.98 AUC, 0.88/0.83–0.97/0.94 sensitivity/specificity, and 0.71–0.90 Youden index for classifying fracture type. When compared with the human groups, the CNN showed superior performance to that of general physicians and orthopedists, similar performance to orthopedists specialized in the shoulder, and the superior performance of the CNN was more marked in complex 3- and 4-part fractures.

Interpretation — The use of artificial intelligence can accurately detect and classify proximal humerus fractures on plain shoulder AP radiographs. Further studies are necessary to determine the feasibility of applying artificial intelligence in the clinic and whether its use could improve care and outcomes compared with current orthopedic assessments.

Proximal humerus fractures are primarily diagnosed using plain radiographs, and the fracture type is determined according to its anatomical location as well as fragmentation and displacement levels. However, since non-orthopedic surgeons or insufficiently experienced orthopedic surgeons are frequently the first doctors to assess fractures, it is not unusual for proximal humerus fractures to be misdiagnosed. In addition, even an experienced orthopedic surgeon can misdiagnose the fracture type due to variable presentation (Mora Guix et al. 2009, Foroohar et al. 2011). Thus, a more efficient and accurate manner of diagnosing and classifying fracture type is of interest.

Deep learning is a branch of artificial intelligence that uses a cascade of many layers of nonlinear processing units to extract features and create transformations and is based on the learning of multiple levels of features or representations of the data (Wang and Summers 2012, Bengio et al. 2013, LeCun et al. 2015). Deep learning comprises a neural network with multiple hidden layers that enhance image recognition accuracy, thereby increasing its versatility for capturing representative features (Shin et al. 2013). Since 2012, deep learning has rapidly become the cutting-edge method of enhancing performance in medial image analysis with the use of convolutional neural networks (CNN), which are well suited for analyzing images, and has led to a decrease in the classification error rate from about 25% in 2011 to 3.6% in 2015 (Russakovsky et al. 2015, Lakhani et al. 2017).

With such success in identifying and classifying images using a deep learning algorithm, there has been interest in applying deep learning to medical image analysis in several fields, including the detection of skin cancer (Esteva et al. 2017), diabetic retinopathy (Gulshan et al. 2016), mammographic lesions (Kooi et al. 2017), and lung nodules (Hua et al. 2015). However, in the field of orthopedic surgery and traumatology, trials are very scarce despite its importance to public health. To our knowledge, only one study (Olczak et al. 2017) has applied deep learning to fracture orthopedics, and reported promising outcomes of deep learning in identifying fracture, laterality, type of view, and body part.

Thus, we aimed to evaluate the diagnostic accuracy of the deep learning algorithm with deep CNN for detecting and classifying proximal humerus fractures using plain anteroposterior (AP) shoulder radiographs. We then compared the results with those of humans.

## Patients and methods

### Dataset

1,891 plain shoulder AP radiographs (1,376 proximal humerus fracture cases and 515 normal shoulders) from 1,891 patients (591 men, 1,300 women; 1,083 from Konkuk University Medical Center, 209 from Kyungpook National University Hospital, 165 from Myungji Hospital, 203 from Kangwon National University Hospital, 41 from the National Police Hospital, 25 from Seoul Saint Mary’s Hospital, and 165 from Wonkwang University Sanbon Hospital) were used as the total dataset in this study. We used only 1 image per person to decrease the overperformance of deep learning by the inclusion of a very similar image of the same patient in each test and training set. The mean age of patients was 65 (24–90) years.

### Fracture classification

To evaluate the performance of fracture classification, we classified the proximal humerus fractures into 4 types based on Neer’s classification, which is the most commonly used classification for the proximal humerus fracture: greater tuberosity, surgical neck, 3-part, and 4-part (Neer et al. 1970). A greater tuberosity fracture was defined as 1 displaced fragment of the greater tuberosity component, and A surgical neck fracture as 1 displaced fragment of the surgical neck component. A 3-part fracture was defined as 2 displaced fragments, while a 4-part fracture was defined as having 3 or more displaced fragments from the proximal humerus. In cases of proximal humerus fractures combined with shoulder dislocation (fracture dislocation type), we used the images after reduction and classified them.

**Figure 1.**
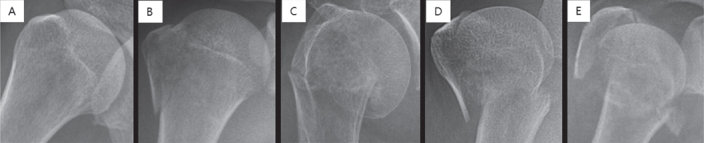
Each shoulder anteroposterior radiograph was manually cropped into a square in which the humeral head and neck are centered such that they comprise approximately 50% of the square image as illustrated above. Images were then resized to 256 × 256 pixels. Examples of normal and each fracture type: (A) normal, (B) greater tuberosity fracture, (C) surgical neck fracture, (D) 3-part fracture, and (E) 4-part fracture.

Each plain shoulder AP radiograph was manually cropped into a square in which the humeral head and neck were centered and constituted approximately 50% of the square image, resized to 256 × 256 pixels, and stored as a JPEG file (Figure 1).

Fracture classification was performed by 2 shoulder orthopedic specialists with 14 and 17 years of experience (SWC and KSO) and 1 radiologist with expertise in musculoskeletal diseases and 15 years of experience (NRK). For cases in which the 3 specialists could not agree, the corresponding CT images were checked (CTs were available for all fractures that failed consensus) and then re-discussed. If consensus still could not be achieved even after the evaluation of the CT image(s), the images were excluded from the dataset (n = 21). 346 cases were ultimately classified as greater tuberosity fractures, 514 cases as surgical neck fractures, 269 cases as 3-part fractures, and 247 cases as 4-part fractures. In addition, 515 cases without proximal humerus fractures were classified as the normal group to evaluate the ability of the CNN to distinguish between normal and fractured shoulders (Figure 1).

### Training of the deep CNN and framework

We used and trained the deep CNN using the training dataset and validated it using the test dataset. The dataset of the 1,891 images was divided into 10 partitions without overlapping images. Among the 10 partitions, 1 partition was used as a test dataset, while all other images were used as training datasets. Thus, for the 10 parts (1 partition = test dataset, the other 9 partitions and remnants = training dataset) 10 experiments were performed, after augmenting the training dataset. The entire training process was then repeated 3 times to adjust for possible deviations in the results. We ran Caffe 9 (http://caffe.berkeleyvision.org/) on Ubuntu 16.04 (https://www.ubuntu.com/download/desktop) with NVIDIA GTX 1070 (CUDA 8.0 and cuDNN 5.1) (https://developer.nvidia.com/cuda-zone and https://developer.nvidia.com/cudnn) and used the open source pre-trained Microsoft ResNet-152 (https://github.com/kaimingHe/deep-residual-networks) as a deep CNN model, and further fine-tuned the pre-trained ResNet model to our proximal humerus fracture datasets (fine-tuning = training using our datasets). The detailed process of the deep CNN training process is shown in the Appendix (see Supplementary data).

### Evaluation of the deep CNN algorithm

After training the deep CNN, we computed the top-1 accuracy. The deep CNN has to answer top-1 (the one with highest probability) to compute the top-1 accuracy, which is the conventional accuracy for the deep CNN answer (top-1) being exactly the expected answer, among 5 choices of normal, greater tuberosity fracture, surgical neck fracture, 3-part fracture, and 4-part fracture. The deep CNN had to find whatever differences it could to make up criteria and define the groups. Then, algorithm performance was measured using the area under the receiver operating curve (AUC) generated by plotting sensitivity versus 1-specificity, which reported the best sensitivity and specificity that maximizes the sum of sensitivity and specificity. In addition, the Youden index (sensitivity + specificity – 1) was calculated. The performance for discerning fractures from normal shoulders and for classifying fractures (the ability to define a certain fracture group (4 fracture group) after excluding normal shoulders from the test set) was evaluated using each value. For fracture type classification, performance was measured only in the fracture images after excluding the normal shoulder images to evaluate the actual performance of fracture classification, thus avoiding the possibility of overfitting of the deep CNN by the inclusion of normal cases that are relatively easy to discern.

### Evaluation of the diagnostic performance of human readers

To compare the performance in diagnosing and classifying the proximal humerus fracture between the CNN and human readers, we provided each reader with the same information as the CNN. The readers consisted of 3 groups of general physicians (n = 28), general orthopedists (n = 11), and orthopedists specialized in shoulders (n = 19). The orthopedic surgeons mainly composed the human readers, as generally an orthopedic surgeon both classifies the fracture on radiographs and takes the decision to operate or not, and then performs surgeries. The 10 parts (181 images each) were converted into 10 image sheets (181 images each) containing the proximal humerus images without explanations (Figure 2, see Supplementary data).

Each reader then received 3 image sheets that were randomly selected using a randomization program (http://www.randomizer.org) and were requested to provide the most probable diagnosis of each image (543 (3 × 181) images) in the form of 1 to 5 (1, normal; 2, greater tuberosity fracture; 3, surgical neck fracture; 4, 3-part fracture; 5, 4-part fracture). We calculated the top-1 accuracy, AUC, sensitivity/specificity, and Youden index for each group of human readers as with the CNN and then compared the values.

### Statistics

All statistical analyses were performed using SPSS 15.0 (SPSS, Inc., Chicago, IL, USA). The receiver operating characteristic curves were generated using a Python script, and each AUC was determined. Descriptive statistics were used to report each value of the top-1 accuracy, AUC, sensitivity/specificity, and Youden index, which was described as a mean and a 95% confidence interval (CI). Comparisons between the CNN and each human group were performed using a one-way analysis of variance, followed by Bonferroni post hoc analysis for multiple comparison with the significance level set at p < 0.05.

### Ethics, funding, and potential conflicts of interest

The study protocol was approved by the local ethics committee (IRB no. KUH1060143) with a waiver of informed consent. This work was supported by Konkuk University in 2017. All authors declare no conflict of interest.

## Results

### Deep learning CNN performance

The top-1 accuracy of the deep learning CNN model in distinguishing between normal and proximal humerus fractured shoulders exhibited more than 95% accuracy (96%, CI 94–97%). Among the proximal humerus fracture cases, the top-1 accuracy of the CNN model for distinguishing each fracture type from the other fracture types was 86% (CI 83–88%) for greater tuberosity fractures, 80% (CI 77–83%) for surgical neck fractures, 65% (CI 59–71%) for 3-part fracture, and 75% (CI 71–79%) for 4-part fractures. The distribution of mispredicted cases in the CNN model is described in Table 1.

**Table 1.** Mispredicted cases in the convolutional neural network model. Values are n (%)

		Types mispredicted as			
Dataset	Normal	Greater tuberosity fracture	Surgical neck fracture	Three- part fracture	Four- part fracture
Normal (n = 1,500)^a^		47 (3)	19 (1)	1 (0)	0 (0)
Greater tuberosity fracture (n = 990)	37 (4)		30 (3)	68 (7)	5 (1)
Surgical neck fracture (n = 1,500)	16 (1)	19 (1)		115 (8)	148 (10)
Three-part fracture (n = 750)	0 (0)	39 (5)	135 (18)		88 (12)
Four-part fracture (n = 690)	2 (0)	1 (0)	98 (14)	70 (10)	

a 50 in each partition x three repetitions x 10 partitions

The deep learning CNN exhibited excellent diagnostic performance with an AUC of 0.996 (CI 0.995–0.998) for discerning normal cases from fracture cases. The CNN accurately classified proximal humerus fractures with an AUC of 0.98 (CI 0.98–0.99) for greater tuberosity fractures, 0.94 (CI 0.93–0.94) for surgical neck fractures, 0.90 (CI 0.89–0.92) for 3-part fractures, and 0.94 (CI 0.93–0.94) for 4-part fractures. At the optimal cutoff point, the mean sensitivity/specificity in the CNN model were 0.99/0.97, 0.97/0.94, 0.90/0.85, 0.88/0.83, and 0.93/0.85 for normal versus all, greater tuberosity, surgical neck, 3-part, and 4-part fractures, respectively. The mean Youden index of each group in the CNN model was as follows: normal, 0.97 (CI 0.96–0.97); greater tuberosity fracture, 0.90 (CI 0.88–0.92); surgical neck fracture, 0.75 (CI 0.73–0.77); 3-part fracture, 0.71 (CI 0.68–0.74); and 4-part fracture, 0.78 (CI 0.77–0.80).

### Comparison between CNN and human reader performance (Tables 2 and 3 and Figure 3)

The CNN showed superior results in diagnosing proximal humerus fractures compared with every human group, although the comparison with the general orthopedist and shoulder orthopedist groups did not reach statistical significance (Table 2).

**Table 2.** Diagnostic accuracy for differentiating proximal humerus fractures from normal shoulders among the CNN and human groups. Values are mean (CI)

	CNN	General physician	General orthopedist	Orthopedists specialized in shoulder	p-value
Top-1 accuracy (%)	96 (94–97)	85 (80–90)^a^	93 (90–96)	93 (87–99)	< 0.001
Sensitivity	0.99 (0.99–1.00)	0.82 (0.78–0.87)^a^	0.93 (0.89–0.97)	0.96 (0.95–0.98)	< 0.001
Specificity	0.97 (0.97–0.98)	0.94 (0.93–0.96)^a^	0.97 (0.96–0.98)	0.98 (0.96–1.00)	0.002
Youden index	0.97 (0.96–0.97)	0.77 (0.72–0.82)^a^	0.90 (0.87–0.94)	0.94 (0.92–0.96)	< 0.001

CNN, convolutional neural network

Youden index was calculated as [sensitivity + specificity – 1].

a Statistically significant in a comparison of CNN and each human group (results from a Bonferroni post hoc analysis)

In addition, the CNN showed the highest performance for classifying proximal humerus fracture types among all fracture types except for greater tuberosity fractures, despite several comparisons with the shoulder orthopedist group not showing statistical significance (Table 3, see Supplementary data).

**Figure 3.**
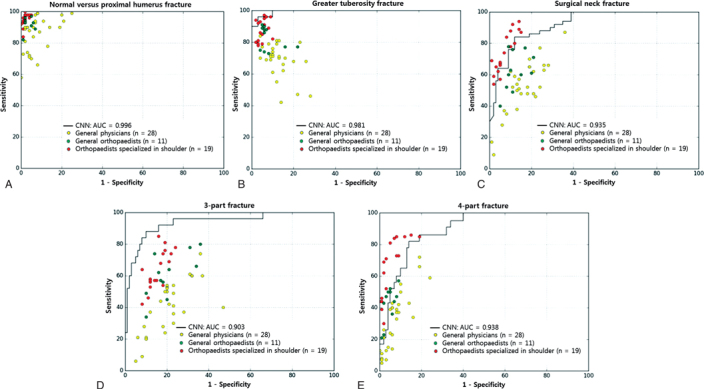
The diagnostic performance between the CNN and each human group was compared using the receiver operating characteristics curves of the CNN and the sensitivity–specificity distribution of each human group to differentiate normal shoulders from proximal humerus fractures (A) and to classify each fracture type: (B) greater tuberosity fracture, (C) surgical neck fracture, (D) 3-part fracture, and (E) 4-part fracture. CNN = convolutional neural network; AUC = area under curve of the receiver operating characteristics curve. The representative receiver operating characteristics curve of the CNN was selected as the curve with the closest AUC value to the average AUC. The dots on the plots represent the sensitivity and specificity of each group (yellow, general physicians; green, general orthopedists; red, orthopedists specialized in the shoulder). All AUCs for the normal shoulder and each fracture type were over 90%. The CNN achieved superior performance at least to a general physician (yellow dot) or to a general orthopedist (green dot), most of whose sensitivity/specificity point lay below the receiver operating characteristic curve of the CNN.

The diagnostic superiority of the CNN compared with the human groups was more marked in 3- and 4-part fractures (Table 3). The CNN was superior to a general physician or general orthopedist on comparing the diagnostic performance of CNN and each human group by overall distribution of the sensitivity/specificity point per person on a receiver operating characteristic curve of the CNN (Figure 3).

## Discussion

In this study, we demonstrate the very high performance of deep learning CNN in distinguishing normal shoulders from proximal humerus fractures. We additionally show promising results for classifying fracture type based on plain shoulder AP radiographs, with the deep learning CNN exhibiting superior performance to that of general physicians and general orthopedists and similar performance to that of the shoulder orthopedists. This indicates the possibility of automated diagnosis and classification of proximal humerus fractures and other fractures or orthopedic diseases diagnosed accurately using plain radiographs. As additional proximal humerus fractures would further enhance the diagnostic performance of the CNN, we think that the deep learning CNN may outperform even the shoulder orthopedists as data accumulate.

Moreover, we found higher performance of CNN, especially in more complex type fractures such as 3- or 4-part fractures, compared with humans, which suggests the superiority of CNN for classifying fractures with various fracture shapes based on plain radiographs because humans have greater difficulty, especially classifying complex fractures, but CNN performs relatively well. Since the number of images for the CNN training was smaller for 3- and 4-part fractures, the results seem more promising. With more training cases of 3- and 4-part fractures, the diagnostic performance of CNN for detecting and classifying complex fractures would improve. The higher performance of CNN for detecting and classifying proximal humerus fractures, especially complex fractures, may in part come from the fact that machine does not suffer from decreases in concentration and is consistent when presented with the same input data (i.e., the CNN will make the same prediction on a specific image every time) unlike humans, who are likely to make an error after a distorted previous experience in fracture classification (humans seem to have a tendency to guess right more often in a typical case but have difficulty when the fracture configuration is a less familiar shape) and through limited concentration. The machine can potentially be trained with an incredible amount of training samples, vastly more than any orthopedist will experience in his/her lifetime, which results in an incomparable possibility of deep learning CNN.

In addition, the diagnostic accuracy of CNN for classifying greater tuberosity fractures was the highest and that of 3-part fracture was the lowest. Greater tuberosity fractures exhibited a distinctive fracture line in the anatomical site of the greater tuberosity with a low variance in the fracture shape among greater tuberosity fractures, whereas all other fracture types in this study have fracture lines in the surgical neck site. We think this anatomical characteristic of the greater tuberosity fracture makes the detection of this fracture type easier with a low error rate. Conversely, the 3-part fracture has a shape between that of a surgical neck fracture and a 4-part fracture. Thus, the CNN seems to confuse more severe 3-part fracture cases with more displacement and angulation with 4-part fractures, while less severe 3-part fracture cases with less displacement and angulation, especially in the greater tuberosity fragments, are confused with surgical neck fractures.

This automated system for detecting and classifying proximal humerus fractures has potential benefits, such as increased accuracy, consistent interpretation, efficiency, near-instantaneous reporting of results, reproducibility, and decreased barriers to access. Since a deep CNN algorithm can have multiple operating points, its sensitivity and specificity can be tuned to match the requirements of specific clinical settings, such as high sensitivity for a screening setting if necessary. With additional data, deep learning will facilitate diagnosis. Furthermore, we believe that the clinical application of deep learning for detection and classification can be expanded to other orthopedic diseases that use radiographs for diagnosis.

Our study has several limitations. First, even though the Neer classification is the most commonly used tool for proximal humerus fracture classification, it has only fair to moderate reliability, and there is no gold standard for proximal humerus fracture classification. Development of a more reliable classification system for proximal humerus fracture could enhance the reliability in classification of the deep learning algorithm. However, the promising result of this study in detecting and classifying proximal humerus fracture by using a deep learning algorithm does not mean that it can be used immediately in clinical practice. This study was not to guide treatment. This study only has the significance that we showed the possibility of the future use of this deep learning algorithm even in the field of orthopedic surgery or traumatology. CNNs that consistently classify fractures could be a giant leap forward. Second, we evaluated the diagnostic performance of CNN based on a cropped single shoulder AP radiograph to keep this project simple, which may not actually reflect a clinically relevant scenario because a fracture evaluation would involve at least 2 radiographs under review. However, the evaluations based on various shoulder radiographs or CT images may enhance the diagnostic performance of CNN as well. Finally, the images were down-sampled to 256 × 256 pixels before they were fed into the network because of the sheer number of parameters inherent to the networks. The diagnostic accuracy may be improved using higher-resolution images. More development on the memory of graphics processing units would allow larger matrix sizes without increasing the training time. In addition, the lossy JPEG compression may influence the image quality. It may be better to use non-lossy compression such as PNG or TIFF.

In conclusion, the use of artificial intelligence can accurately detect and classify proximal humerus fractures on plain shoulder AP radiographs. Further studies are necessary to determine the feasibility of applying artificial intelligence in the clinic and whether its use could improve care and outcomes compared with current orthopedic assessments.

### Supplementary data

Figure 2, Table 3 and the Appendix are available as supplementary data in the online version of this article, http://dx.doi.org/10.1080/17453674.2018.1453714

Supervision: SWC, YK. Conception and design: SWC, SSH, JWL, K-SO, NRK, YK. Acquisition of data: SWC, JWL, K-SO, JPY, JYK, SHM, JK, H-JL, Y-MN. Analysis and interpretation of data: SWC, SSH, NRK, JPY, JYK, SHM, JK, H-JL, Y-MN, YK.

*Acta* thanks Max Gordon and other anonymous reviewers for help with peer review of this study.

## Supplementary Material

IORT_A_1453714_SUPP.PDF
