# Supplementary material for: Automated detection and classification of the proximal humerus fracture by using deep learning algorithm
Source: Acta Orthop. 2018 Mar 26;89(4):468–73. doi: 10.1080/17453674.2018.1453714 (PMC6066766; doi:10.1080/17453674.2018.1453714)
Supplement: IORT_A_1453714_SUPP.PDF [file IORT_A_1453714_SM7367.pdf]

Supplementary data

Appendix

Training of the deep CNN and framework

The dataset of the 1,891 images was divided into 10 partitions without overlapping images. Each partition was composed of randomly selected images including 50 normal, 33 greater tuberosity fracture, 50 surgical neck fracture, 25 3-part fracture, and 23 4-part fracture (total, 181 images). This corresponding number of each image group (50, 33, 50, 25, and 23, respectively) was graded on a scale of 1 to 10 (approximately 1/10 of the total image number of each group [515, 346, 514, 269, and 247, respectively]). The rest of the 81 images (1,891 images – 181 images × 10 partitions) were later added to a training set.

Among the 10 partitions, 1 partition was used as a test dataset, while all other images were used as training datasets. Each partition subsequently acted as a test dataset. Since the initial values of the outer layer of the pre-trained model were randomly set during fine-tuning, the entire training process was repeated 3 times to adjust for possible deviations in the results. The training dataset was augmented (shifting, scale transformations, and rotation) to account for small datasets. Using shifting (in the upward, downward, left, and right directions) and scale transformation (15% magnification), we multiplied the dataset 6 times. Then, applying the rotation method (90°, 180°, and 270° rotation) to this multiplied dataset, we finally multiplied the dataset 24 times. Thus, after augmentation, the training dataset consisted of more than 40,000 images (1,710 × 24) at a time.

We used Caffe (http://caffe.berkeleyvision.org), the most widely used open-source deep learning framework developed by the Berkeley Vision and Learning Center. We ran Caffe on Ubuntu 16.04 with NVIDIA GTX 1070 (CUDA 8.0 and cuDNN 5.1) and used Microsoft ResNet-152 as a deep CNN model. We further fine-tuned the pre-trained ResNet model to our proximal humerus fracture datasets to utilize the pre-trained earlier layers that contain more generic features while maintaining earlier layers as relatively fixed and updating later layers of the network (base_lr: 0.0001; max: 3 epochs; step: 2 epochs; gamma: 0.1; weight_decay: 0.00001; train_batch_size: 24; 1 epoch indicates the number of training iterations in which the neural network reviewed an entire training set).

Table 3. Comparison of the performance of classifying proximal humerus fracture types of CNN versus each human group. Values are mean (CI)

Performance of classifying each fracture type	CNN	General physician	General orthopedist	Orthopedists specialized in shoulder	p-value
Greater tuberosity fracture					
Top-1 accuracy (%)	86 (83–88)	82 (78–86)	90 (85–94)	93 (91–96) [a]	< 0.001
Sensitivity	0.97 (0.95–0.98)	0.66 (0.61–0.71) [a]	0.80 (0.72–0.88) [a]	0.88 (0.85–0.91) [a]	< 0.001
Specificity	0.94 (0.92–0.95)	0.94 (0.93–0.96)	0.97 (0.95–0.98)	0.98 (0.97–0.99) [a]	< 0.001
Youden index	0.90 (0.88–0.92)	0.60 (0.55–0.66) [a]	0.76 (0.69–0.84) [a]	0.86 (0.83–0.88)	< 0.001
Surgical neck fracture					
Top-1 accuracy (%)	80 (77–83)	56 (49–64) [a]	64 (55–73) [a]	76 (70–82)	< 0.001
Sensitivity	0.90 (0.87–0.93)	0.69 (0.65–0.72) [a]	0.78 (0.73–0.82) [a]	0.88 (0.86–0.91)	< 0.001
Specificity	0.85 (0.83–0.88)	0.76 (0.73–0.79) [a]	0.80 (0.76–0.84)	0.87 (0.84–0.90)	< 0.001
Youden index	0.75 (0.73–0.77)	0.45 (0.41–0.48) [a]	0.58 (0.52–0.63) [a]	0.76 (0.73–0.77)	< 0.001
Three-part fracture					
Top-1 accuracy (%)	65 (59–71)	42 (35–48) [a]	62 (54–71)	65 (59–71)	< 0.001
Sensitivity	0.88 (0.86–0.91)	0.33 (0.30–0.35) [a]	0.44 (0.38–0.50) [a]	0.52 (0.49–0.55) [a]	< 0.001
Specificity	0.83 (0.80–0.85)	0.84 (0.83–0.86)	0.90 (0.88–0.92) [a]	0.91 (0.90–0.92) [a]	< 0.001
Youden index	0.71 (0.68–0.74)	0.17 (0.13–0.20 [a]	0.34 (0.27–0.41) [a]	0.43 (0.40–0.47) [a]	< 0.001
Four-part fracture					
Top-1 accuracy (%)	7 5 (71–79)	32 (25–39) [a]	43 (36–51) [a]	65 (56–74)	< 0.001
Sensitivity	0.93 (0.91–0.95)	0.56 (0.49–0.63) [a]	0.70 (0.63–0.77) [a]	0.79 (0.72–0.85) [a]	< 0.001
Specificity	0.85 (0.83–0.88)	0.86 (0.85–0.87)	0.89 (0.87–0.90)	0.93 (0.91–0.94) [a]	< 0.001
Youden index	0.78 (0.77–0.80)	0.42 (0.36–0.48) [a]	0.59 (0.52–0.65) [a]	0.71 (0.66–0.77)	< 0.001

CNN, convolutional neural network
Youden index was calculated as [sensitivity + specificity – 1].
[a] Statistically significant in a comparison of CNN and each human group (results of a Bonferroni post hoc analysis)

 Acta Orthopaedica 2018; 89 (DOI: 10.1080/17453674.2018.1453714)

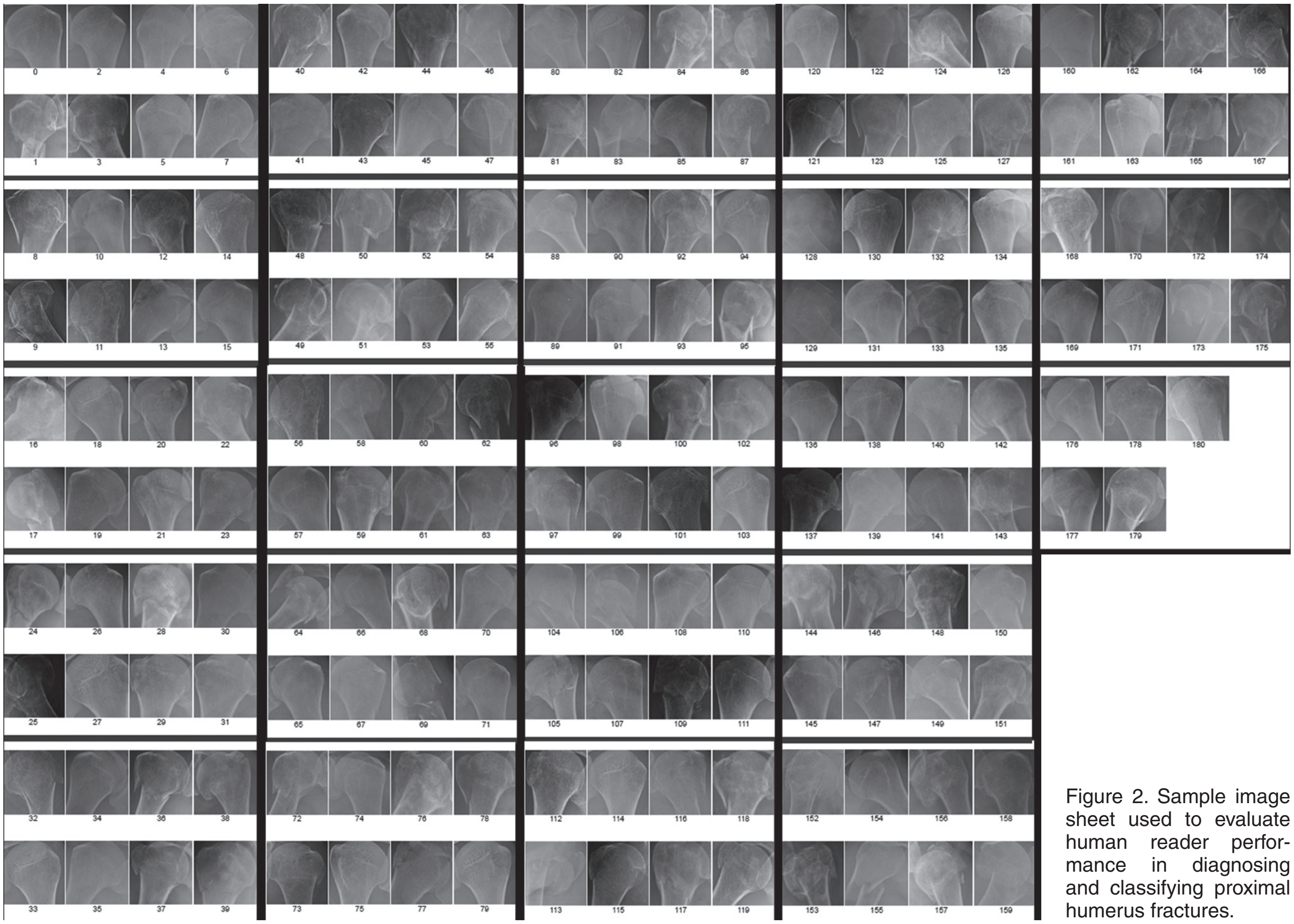

Figure 2. Sample image sheet used to evaluate human reader performance in diagnosing and classifying proximal humerus fractures.
